# Dento‐Skeletal and Upper Airways Effects of Class III Intermaxillary Elastics in Growing Patients: A Retrospective Controlled Study

**DOI:** 10.1002/cre2.70491

**Published:** 2026-10-25

**Authors:** Michele d'Attilio, Gianluca Rosa, Salvo Luca Gallo, Giuliano Ascani, Antonino Peluso

**Affiliations:** ^1^ Department of Innovative Technologies in Medicine & Dentistry University “G. d'Annunzio” Chieti‐Pescara Chieti Italy; ^2^ Department of Maxillofacial Surgery Spirito Santo Hospital Pescara Italy; ^3^ Telematic University Leonardo da Vinci UNIDAV Torrevecchia Teatina Chieti Italy

**Keywords:** cephalometry, Class III malocclusion, early orthodontic treatment, intermaxillary elastics, upper airway

## Abstract

**Objective:**

To evaluate the dento‐skeletal and upper airway effects of a fully intraoral fixed appliance activated with Class III intermaxillary elastics in growing patients with skeletal Class III malocclusion, compared with an untreated matched control group.

**Materials and Methods:**

This retrospective controlled study included 18 growing patients treated with an intraoral appliance anchored to molar bands in both arches activated with intermaxillary elastics. An untreated control group of 18 subjects matched for skeletal disharmony, sex, chronological age, and observation interval was selected from the Burlington Growth Study (AAOF) database. Lateral cephalograms were obtained before (T0) and at the end of treatment (T1) and analyzed using the Steiner and Kirjavainen analyses. ANB and Wits appraisal were defined as primary outcomes.

**Results:**

No statistically significant baseline differences were found between groups. The study group showed a statistically significant improvement of the sagittal intermaxillary relationship as measured by the Wits appraisal (Δ = +4.60 mm), whereas the between‐group difference in the ANB angle did not reach statistical significance (Δ = +0.59°). SNB remained essentially stable in the treated group (Δ = +0.01°) compared with a slight forward repositioning in controls (Δ = +0.78°). All vertical parameters showed no significant between‐group differences. A non‐significant trend toward upper incisor proclination was observed, while IMPA remained essentially stable (Δ = +1.58°). Airway changes were limited and topographically selective: significant increases were observed at the nasopharyngeal level and in soft palate length, with no significant changes at the velopharyngeal or oropharyngeal levels.

**Conclusions:**

The present study suggests that a fully intraoral fixed appliance activated with Class III intermaxillary elastics may represent a possible early treatment option in growing Class III patients. These preliminary findings should be confirmed by prospective controlled studies with larger samples, three‐dimensional airway assessment and long‐term follow‐up.

## 1 Introduction

According to Angle's classification, class III malocclusion is defined as a condition where the mandibular first molar is positioned mesially relative to maxillary first molar (Proffit and Fields 2000), this relationship is often associated with an anterior crossbite.

Previous studies suggest that the etiology of Class III malocclusion is due to a combination of hereditary and environmental factor (Xue et al. 2010; Otero et al. 2014) which may also affect the long‐term stability of the treatment (Masucci et al. 2011; Westwood et al. 2003).

The prevalence of Class III malocclusion differs across regions, ranging from 0.84% to 2.68% in the Americas, 0.59% to 4.6% in Africa, 1.21% to 2.75% in Europe, and 3.91% to 6.46% in Asia (Alhammadi et al. 2018).

Considering the natural tendency for skeletal class III to worsen over time (Wolfe et al. 2011), early diagnosis and intervention of class III malocclusion is crucial for a normal oral and maxilla‐facial development. Moreover, it can reduce the need for treatment during the permanent dentition stage and help prevent the necessity for later surgical intervention (Mandall et al. 2016; Zhou et al. 2025; Ahn et al. 2012).

Several orthopedic approaches have been proposed for the early management of Class III malocclusion, and they can be broadly grouped into extraoral and intraoral strategies (Rongo et al. 2017).

Among extraoral devices, facemask (FM) therapy represents the most commonly used treatment, and it can be performed in conjunction or not with a rapid palatal expander. A systematic review and meta‐analysis by Cordasco et al. (2014). conclude that in the short term, FM therapy produce forward movement of the maxilla with a backward movement of the mandible, clockwise rotation of the mandibular plane and counterclockwise rotation of the maxillary plane. Also, rapid palatal expansion (RPE) seems not to improve the effectiveness of FM (Cordasco et al. 2014). More recently, another systematic review and meta‐analysis from Rutili et al. (2023). has compared the effect of FM with and without skeletal anchorage. The result from the latter study suggests that skeletal anchored FM is equally effective as the dental anchored FM in short‐term, but with less proclination of upper central incisors.

The SEC III protocol, introduced by Ferro et al. in 1980 (Ferro et al. 2003) combines two occlusal splints, Class III elastics, and an extraoral chin‐cup to control the vertical dimension, and has shown favorable effects on Wits appraisal and ANB angle when compared with RPE/FM (Perillo et al. 2016; Nucci et al. 2021).

The main issue with the use of extraoral devices is the poor patients' compliance. To overcome this issue, authors have tried to develop intraoral appliances to correct class III treatment. The Ertty Gap III system, for example, combines an upper Hyrax‐type expander with anterior and posterior screws and a lower lip bumper anchored to the first permanent molars, and is activated with heavy Class III elastics with the aim of stimulating the premaxillary suture and producing counter‐clockwise rotation of the premaxilla.

The aim of this intraoral system is to stimulate the premaxillary suture to increase maxillary sagittal projection through counterclockwise rotation of the premaxilla, integrated with Class III elastics (which attempt to limit mandibular sagittal growth) rotating the mandibular plane clockwise and the occlusal plane counterclockwise (Análisis de los fundamentos biológicos del Sistema Ertty Gap III® n.d.). Despite its biological plausibility and clinical appeal, the dento‐skeletal effects of this appliance have been only partially documented, and high‐quality controlled evidence is currently lacking.

Beyond the dento‐skeletal district, the skeletal changes induced by Class III orthopedic treatment may also influence the morphology of the upper airway. A study by Havakeshian et al. (2020). indicates modest improvements, especially in the nasopharyngeal and velopharyngeal regions, after maxillary traction with FM.

Switching from dentally anchored to skeletally anchored FM therapy, it tends to be associated with greater improvements in pharyngeal dimensions, although the results are not uniform across all region (Seo and Han 2017).

A systematic review by Adel et al. concluded that despite favorable trends, the overall evidence is still not strong (low number of studies and low quality of evidence) to support a significant increase in airway size following bone‐anchored maxillary protraction (BAMP) (Adel et al. 2023).

Airway changes following intermaxillary Class III traction have been explored with removable splint‐based appliances such as the modified tandem traction bow appliance (MTTBA), which showed an enlargement of the oropharyngeal region (Kaygisiz et al. 2022); however, this study did not include an untreated control group. To the best of our knowledge, whether comparable airway changes can be achieved with a fixed, fully intraoral, tooth‐borne appliance activated with Class III elastics has not yet been investigated against an untreated control group.

Based on these considerations, the present retrospective controlled study was designed to address if a fixed intraoral appliance activated by Class III intermaxillary elastics in growing patient compared with an untreated matched control group, produce significant dento‐skeletal and upper airway changes.

Accordingly, the primary objective of this study was to evaluate the cephalometric dento‐skeletal effects of the appliance, while the secondary objective was to assess concomitant changes in upper airway dimensions.

## 2 Materials and Methods

### 2.1 Sample Size Calculation

The present study was designed as a retrospective study.

The sample size was calculated considering *α* = 0.05, power = 80% and effect size of 1 with two primary clinical outcomes: ANB angle and Wits appraisal.

For the ANB angle, a minimum clinically relevant mean difference of 2° between the treated and untreated groups was assumed, with a common standard deviation of 2°.

For the Wits appraisal, a minimum clinically relevant mean difference of 2 mm was assumed, with a common standard deviation of 2 mm.

Based on these parameters, a sample size of at least 34 subjects (17 subjects per group) was required to detect a statistically significant between‐group difference for both primary outcomes. To compensate for possible incomplete records or methodological dropouts, the study group was expanded to 18 subjects, and matched with an equal number of untreated controls obtained from the Burlington Growth Study database.

### 2.2 Patient Recruitment and Treatment

A total of 33 patients treated with intraoral appliance activated with Class III elastics at the orthodontic department of the University of Studies “G. d'Annunzio” Chieti‐Pescara between 2018 and 2024 were retrospectively identified from the clinical archive and screened for eligibility according to a consecutive sampling approach.

The inclusion criteria were the following:

− Chronological age between 6 and 13 years.
− Skeletal maturation stage between CS1 and CS3 according to the cervical vertebral maturation (CVM) (Baccetti et al. 2005).
− Caucasian ethnicity (self‐reported by parents), to ensure ethnic homogeneity with the AAOF Burlington control sample.
− Early or late mixed dentition, defined as the simultaneous presence of deciduous and permanent teeth in the same arch.
− Skeletal class III malocclusion, defined as a pre‐treatment Wits appraisal ≤ −2 mm.
− Class III molar relationship, defined as either a mesial step relationship of the deciduous second molars or a full class III relationship of the permanent first molars, depending on the dentition stage of the patient.

The exclusion criteria were the following:

− Previous orthodontic or orthopedic treatment.
− Craniofacial syndromes or cleft lip and/or palate.
− Systemic diseases or pharmacological therapies known to affect craniofacial growth (e.g., endocrine disorders, juvenile idiopathic arthritis, chronic systemic corticosteroid therapy).
− Dental developmental anomalies (e.g., agenesis, supernumerary teeth, ectopic eruption, impacted teeth involving the molar or incisor regions).
− Temporomandibular joint disorders.
− History of facial trauma.

From the total sample, 15 patients were excluded for the following reasons: five for incomplete clinical records, three for previous orthodontic treatment, three for non‐Caucasian ethnicity, two for dental developmental anomalies, one for craniofacial syndrome, and 1 for a systemic disease affecting craniofacial growth. The final study group comprised 18 subjects (13 female and 5 male) aged between 6 and 13 years.

Informed consent was obtained from all the patients' parents involved in the study and the study was conducted in accordance with the Declaration of Helsinki and Good Clinical Practice guidelines.

All the study group patients were treated with a fixed intraoral appliance with oral and vestibular SS wires (1 mm diameter oral and 1.2 mm vestibular) anchored to molar bands. The appliance was placed both in maxillary and mandibular arches and activated with class III intermaxillary elastics (Figures 1 and 2).

**Figure 1:**
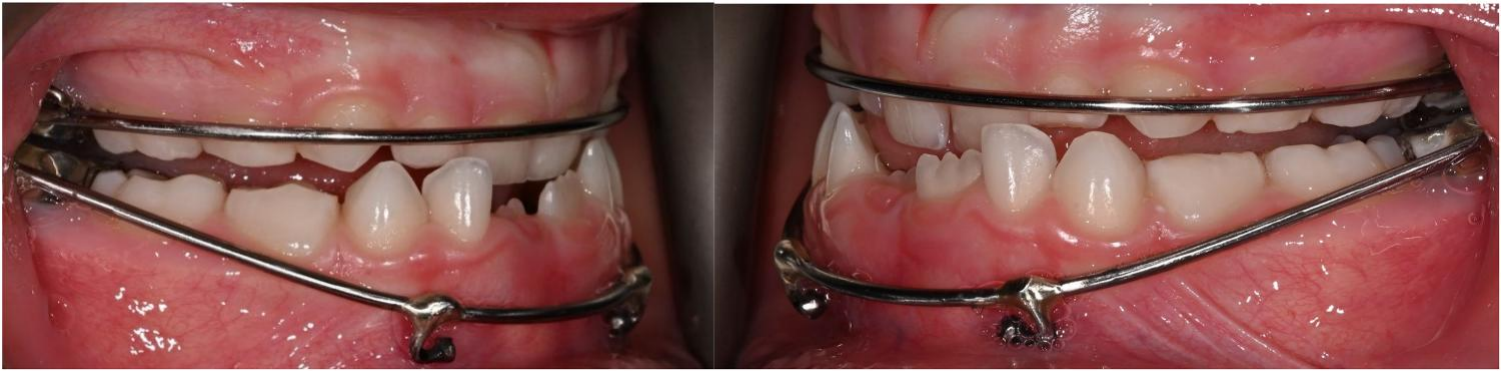
Frontal and lateral view of the appliance.

**Figure 2:**
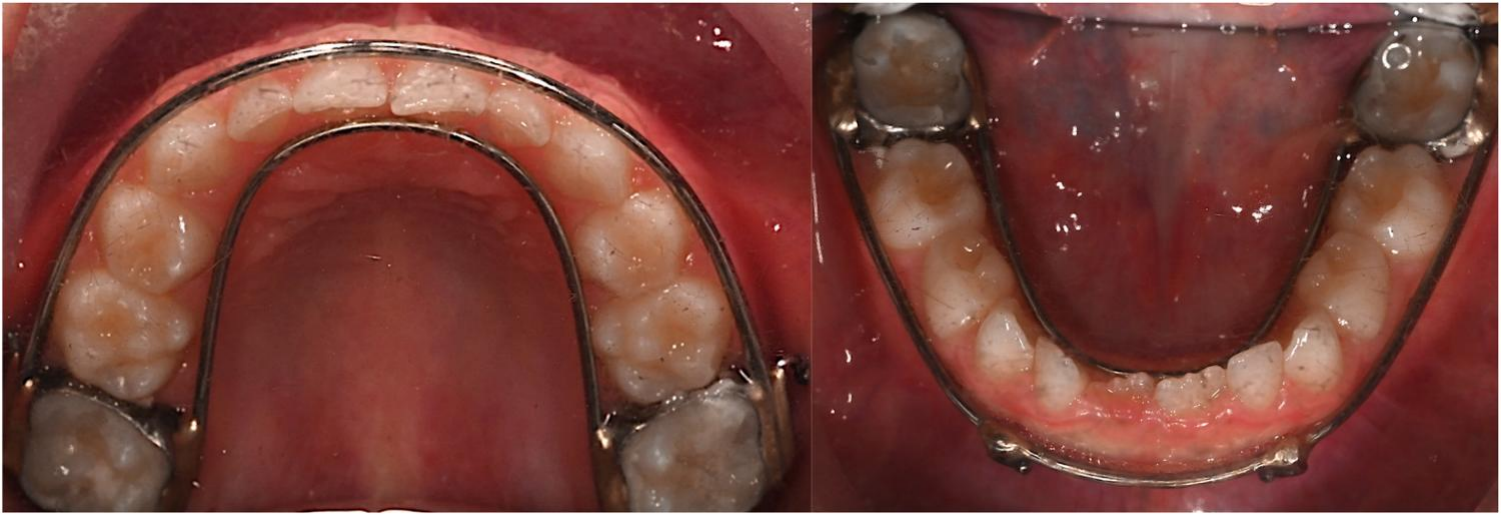
Occlusal view of the appliance.

The appliance was placed on first molars or second deciduous molars based on Half Pulp Chamber line (HPC line) (Primozic et al. 2021) and all the patients were instructed to wear 3/16 4.5 oz elastics for 18 h per day.

Lateral cephalograms were obtained before (T0) and at the end of the treatment (T1).

The active phase of the treatment ended when a class II molar relationship was achieved. In the retention phase the patients were ask to use the elastics only during the night.

A control group of 18 untreated subjects (13 female and 5 male) was selected from the Burlington Growth Study database of the American Association of Orthodontists Foundation (AAOF) Craniofacial Growth Legacy Collection Project (www.aaoflegacycollection.org).

T0 and T1 cephalograms of control subjects were therefore selected from the longitudinal records available in the database. A matching procedure was performed: for each subject in the study group, an untreated subject was selected from the database such that the two groups were comparable for:

– Skeletal disharmony
– Gender
– Chronological age at T0 and T1
– Mean duration of the observational interval

Comparability between the two groups at baseline was subsequently confirmed by statistical analysis.

### 2.3 Cephalometric Analysis

Cephalometric analysis was performed with OrisCeph3 software (OrisLine; Elite Computer Italia s.r.l., Milano, Italy). In order to evaluate skeletal and airway parameters, lateral cephalogram were analyzed using the Steiner and Kirjavainen cephalometric analyses (Kirjavainen and Kirjavainen 2007; Steiner 1960).

All the measured variables are shown in Tables 1 and 2 and illustrated in Figures 3A,B and 4A,B.

**Table 1:** Cephalometric skeletal variables.

Index	Normal value and SD	Description	Unit
SNA	82 ± 2	Angle formed between points S, N, and A	Degree
SNB	80 ± 2	Angle formed between points S, N, and B	Degree
ANB	2 ± 2	Angle formed between points A, N, and B	Degree
Wits apprisal	0 ± 2	Distance between points A and B reported orthogonally on the Ricketts occlusal plane	mm
SN^GoGn	32 ± 5	Angle between the cranial base (SN) and the geometric mandibular plane (GoGn)	Degree
FMA	26 ± 5	Angle between the Frankfort plane and the anatomical mandibular plane (GoMe)	Degree
MM	27 ± 5	Angle between the bispinal line (ANS‐PNS) and the anatomical mandibular plane (GoMe)	Degree
I+/ANS‐PNS	Hypodivergent: 113 ± 2	Angle between the upper incisor and the bispinal plane	Degree
	Normodivergent: 110 ± 2		
	Hyperdivergent: 107 ± 2		
IMPA	Hypodivergent: 93 ± 3	Angle between the lower incisor and the anatomical mandibular plane	Degree
	Normodivergent: 90 ± 3		
	Hyperdivergent: 87 ± 3		
Gonial angle	120 ± 5	Angle formed between points Ar, Go, and Me	Degree
Superior gonial angle	50 ± 2	Superior angle formed from the total goniac angle by drawing a straight line passing through Go and N	Degree
Inferior gonial angle	70 ± 3	Lower angle formed from the total goniac angle by drawing a straight line passing through Go and N	Degree

**Table 2:** Kirjavainen cephalometric analysis.

Index	Description	Unit
AA–PNS distance	The distance of the most anterior point of atlas vertebra (AA) to posterior nasal spine (PNS)	mm
ve–pve distance	The distance of the closest point of soft palate to the posterior pharyngeal wall (velum palatinum, ve) to the horizontal counterpoint on the posterior pharyngeal wall (pve)	mm
Pas‐ppas distance	The distance of the intersection points on anterior and posterior pharyngeal wall of the line from supramentale (B) to gonion (Go)	mm
Ph‐pph distance	The distance of horizontal counterpoints on anterior and posterior pharyngeal wall in oropharynx at its narrowest area	mm
PNS‐p distance	The distance of posterior nasal spine (PNS) to tip of soft palate (p)	mm
tt‐eb length	Tongue length. The distance from anterior point of tip of tongue (tt) to the base of epiglottis (eb)	mm
Th height	Tongue height. The perpendicular distance of superior point of tongue (th) bellow posterior nasal spine (PNS) to line from the tongue tip (tt) to the intersection point of tongue and mandibular border (tg)	mm
H‐H' distance	The distance from the most anterior and superior point of hyoid bone (H) perpendicular to mandibular plane (MP)	mm
H‐C3ai distance	Hyoidale (H). The perpendicular distance from the most anterior and superior point of hyoid bone to perpendicular line from C3ai to HPT	mm

**Figure 3:**
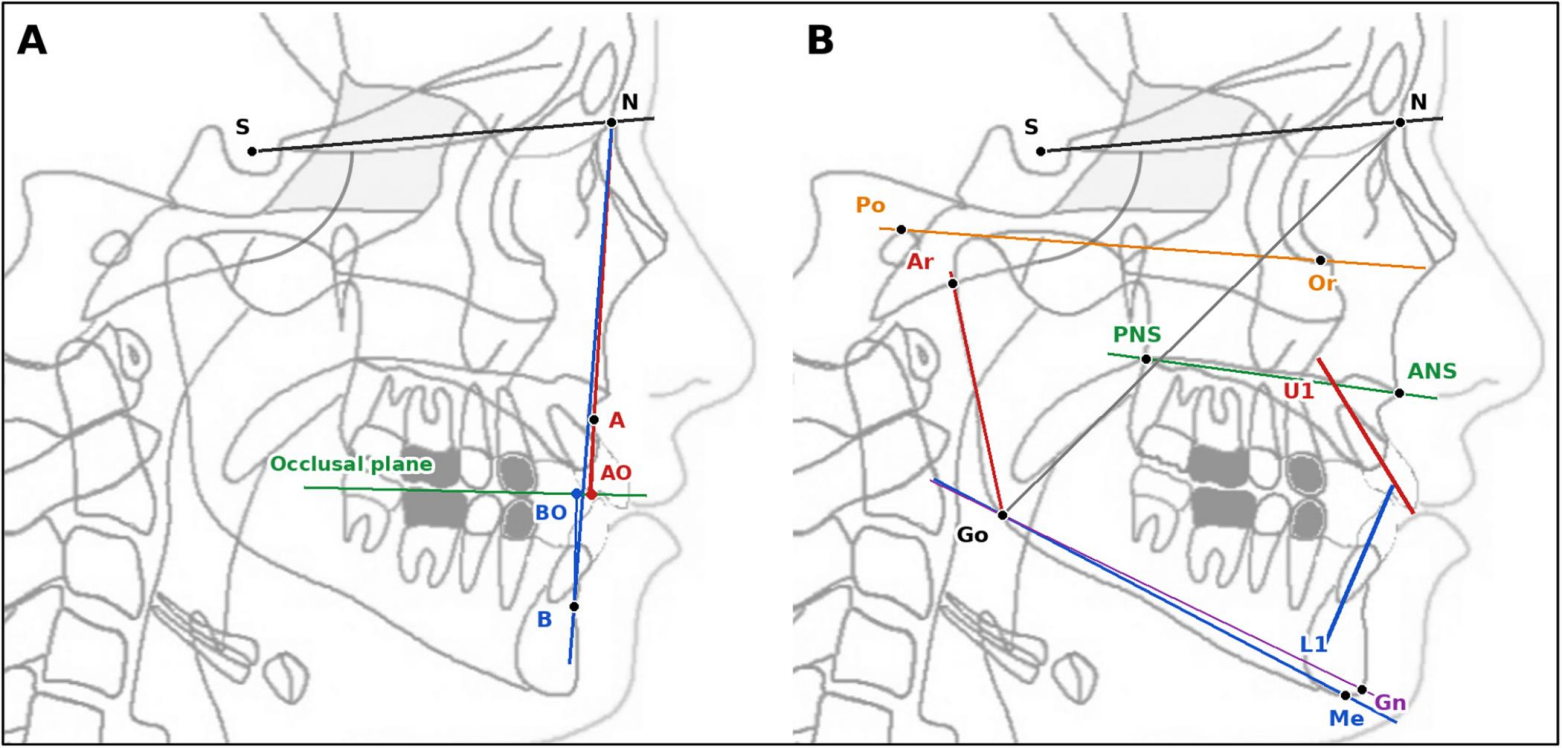
Schematic representation of the skeletal and dental cephalometric measurements. (A) sagittal skeletal measurements. (B) vertical skeletal and dental measurements.

**Figure 4:**
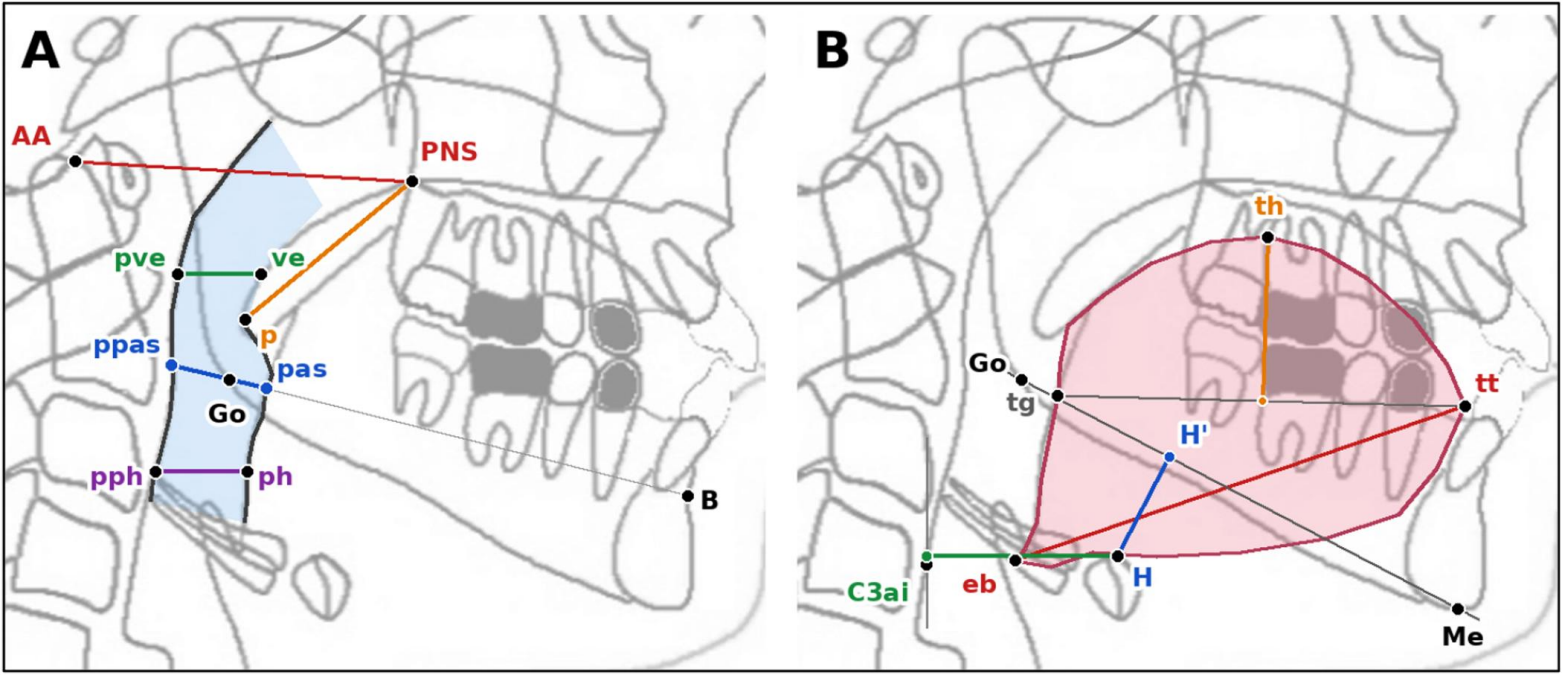
Schematic representation of the airway, tongue, and hyoid bone measurements. (A) upper airway measurements. (B) tongue and hyoid bone measurements.

The measurements were performed on T0 and T1 cephalogram.

### 2.4 Method Error

Method error was assessed by retracing 10 randomly selected cephalograms from the eligible patients at T0 and T1. The same examiner retraced the same set of landmark after a wash‐out period of 4 weeks. The method error for all measurements was calculated using Dahlberg's formula (Statistical Methods for Medical and Biological Students 1940). The systematic error was assessed by using a pair *t*‐test and comparing the first and the second measurement (*p *< 0.05).

### 2.5 Statistics

Statistical analysis was performed using Jamovi Software version 2.6.44.

The ANB angle and the Wits appraisal were defined as primary outcomes. The remaining skeletal cephalometric variables (Table 1) were considered secondary outcomes, intended to characterize the global dento‐skeletal response to treatment. The airway and soft‐tissue variables (Table 2) were considered exploratory outcomes, intended to describe potential concomitant changes of the upper airway and surrounding soft tissues.

The following comparisons were made:

– Baseline (T0) comparison between the study group and the control group.
– Comparison of changes between T1 and T0 between the study group and the control group.

Normality distribution was assessed using the Shapiro–Wilk test and depending on the variable's distribution type (normally distributed or not), a parametric test (Student's *t*‐test) or a nonparametric test (Mann–Whitney test) was used, respectively.

The level of statistical significance was set at *p*‐value < 0.05.

## 3 Results

### 3.1 Method Error

The method error was small for all variables, ranging between 0.28 and 0.62 mm for linear measurement and from 0.48° and 0.91° for angular measurement. No systematic error for any measurement (*p *> 0.05). No systematic error was detected for any measurement (student's *t*‐test: *p* > 0.05).

### 3.2 Baseline Comparison

Tables 3 and 4 present descriptive statistics and comparison at baseline for case and control group. No statistically significant differences were found between the two groups for any cephalometric variables.

**Table 3:** Skeletal cephalometric baseline comparison between study group and control group.

Variables	Study group (Mean ± SD)	IC 95% study group	Control group (Mean ± SD)	IC 95% control group	*p* value
SNA	83.36 ± 3.43	81.65–85.07	81.76 ± 4.71	79.42–84.10	0.252
SNB	80.57 ± 3.66	78.75–82.39	79.53 ± 4.32	77.38–81.68	0.444
ANB	2.78 ± 2.93	1.32–4.24	2.23 ± 2.09	1.19–3.27	0.517
Wits apprisal	–6.59 ± 7.64	−10.39 to −2.79	−3.32 ± 3.01	−4.82 to −1.82	0.105
Sn^GoMe	33.93 ± 5.42	31.23–36.63	34.40 ± 5.40	31.71–37.09	0.795
FMA	25.65 ± 5.36	22.98–28.32	25.37 ± 6.31	22.23–28.51	0.885
MM	25.37 ± 4.46	23.15–27.59	26.17 ± 5.41	23.48–28.86	0.634
I+/ANS‐PNS	112.49 ± 5.44	109.78–115.20	110.62 ± 8.16	106.56–114.68	0.425
IMPA	91.99 ± 7.01	88.50–95.48	89.38 ± 4.66	87.06–91.70	0.197
Gonial angle	130.51 ± 4.32	128.36–132.66	128.13 ± 6.54	124.88–131.38	0.207

**Table 4:** Kirjavainen baseline comparison between study group and control group.

Variables	Study group (Mean ± SD)	IC 95% study group	Control group (Mean ± SD)	IC 95% control group	*p* value
AA‐PNS distance	31.71 ± 2.0	30.71–32.7	32.38 ± 2.1	31.34–33.43	0.6617
ve‐pve distance	8.54 ± 1.9	7.6–9.49	9.3 ± 2.22	8.2–10.4	0.704
pas‐ppas distance	46.01 ± 12.59	39.74–52.27	47.29 ± 15.78	39.44–55.13	0.6348
ph‐pph distance	10.91 ± 2.7	9.56–12.25	11.88 ± 3.02	10.38–13.39	0.3667
PNS‐p distance	27.65 ± 2.84	26.24–29.06	28.74 ± 4.37	26.57–30.91	0.2017
tt‐eb length	61.43 ± 4.54	59.17–63.68	64.34 ± 5.6	61.55–67.12	0.4194
Th height	11.48 ± 2.52	10.23–12.73	10.34 ± 2.24	9.23–11.46	0.0657
H‐H' distance	9.93 ± 4.5	7.7–12.17	9.87 ± 2.85	8.46–11.29	0.3979
H‐C3ai distance	−11.99 ± 33.85	−28.82 to 4.84	−4.17 ± 4.37	−6.34 to −2.0	0.635

### 3.3 Dento‐Skeletal Changes

The comparisons of changes between *T*_0_ and *T*_1_ are summarized in Table 5.

**Table 5:** Skeletal cephalometric changes comparison between study group and control group.

Variables	Δ Study group (Mean ± SD)	IC 95% study group	Δ Control group (Mean ± SD)	IC 95% control group	Δ (study group – Control group)	*p* value
SNA	−0.14 ± 4.11	−2.19 to 1.90	0.04 ± 2.56	−1.24 to 1.31	−0.18	0.873
SNB	0.01 ± 3.39	−1.68 to 1.70	0.78 ± 2.30	−0.36 to 1.93	−0.77	0.430
ANB	−0.17 ± 3.05	−1.69 to 1.35	−0.76 ± 1.73	−1.62 to 0.11	0.59	0.481
Wits Apprisal	3.13 ± 8.08	−0.89 to 7.14	−1.47 ± 3.47	−3.20 to 0.25	4.60	**0.037**
Sn^GoMe	−0.44 ± 4.70	−2.78 to 1.90	−0.93 ± 5.11	−3.47 to 1.61	0.49	0.764
FMA	0.96 ± 5.02	−1.53 to 3.46	0.44 ± 5.08	−2.08 to 2.97	0.52	0.761
MM	−2.24 ± 5.56	−5.01 to 0.52	−0.70 ± 4.11	−2.74 to 1.34	−1.54	0.350
I+/ANS‐PNS	4.44 ± 6.72	1.10–7.79	1.58 ± 6.52	−1.66 to 4.83	2.86	0.204
IMPA	−0.08 ± 6.20	−3.16 to 3.00	−1.66 ± 5.63	−4.46 to 1.14	1.58	0.428
Gonial Angle	−2.90 ± 5.97	−5.87 to 0.07	−1.71 ± 5.01	−4.20 to 0.78	−1.19	0.522

*Note:* Bold values indicate statistical significance (*p* < 0.05).

The study group exhibited a statistically significant increase in the Wits appraisal compared with controls (Δ = +4.60 mm, *p* = 0.037). The ANB angle showed a small, non‐significant between‐group difference (Δ = +0.59°, *p* = 0.481). The SNA angle remained essentially unchanged in both groups (Δ = −0.18°, *p* = 0.873), and SNB showed a non‐significant difference (Δ = −0.77°, *p* = 0.430), with a slight forward repositioning of the mandible observed in the control group (+0.78°) but not in the study group (+0.01°).

Regarding vertical parameters, no significant differences were observed between groups for SN^GoGn (Δ = +0.49°, *p* = 0.764), FMA (Δ = +0.52°, *p* = 0.761), or MM angles (Δ = −1.54°, *p* = 0.350).

In the dentoalveolar region, upper incisors showed a tendency toward labial inclination in the study group (I+/ANS‐PNS Δ = +2.86°, *p* = 0.204) and no significant between‐group differences were observed for IMPA (Δ = +1.58°, *p* = 0.428).

### 3.4 Airway and Soft Tissue Changes

Airway and soft tissue changes between *T*_0_ and *T*_1_ are reported in Table 6.

**Table 6:** Kirjavainen cephalometric changes comparison between study group and control group.

Variables	Δ Study group (Mean ± SD)	IC 95% study group	Δ Control group (Mean ± SD)	IC 95% control group	Δ (study group – control group)	*p* value
AA‐PNS distance	0.68 ± 2.26	−0.44 to 1.8	−1.91 ± 4.66	−4.23 to 0.41	2.59	**0.0442**
ve‐pve distance	0.76 ± 2.06	−0.27 to 1.78	−0.44 ± 2.38	−1.62 to 0.74	1.2	0.115
pas‐ppas distance	1.28 ± 21.11	−9.22 to 11.78	−6.48 ± 20.41	−16.63 to 3.66	7.76	0.27
ph‐pph distance	0.98 ± 3.96	−0.99 to 2.95	−0.93 ± 2.99	−2.42 to 0.56	1.91	0.112
PNS‐p distance	1.09 ± 4.68	−1.24 to 3.42	−2.69 ± 4.83	−5.09 to −0.29	3.78	**0.0229**
tt‐eb length	2.91 ± 6.59	−0.37 to 6.19	−1.79 ± 10.82	−7.17 to 3.59	4.7	0.127
Th height	−1.13 ± 3.43	−2.84 to 0.57	−1.39 ± 2.06	−2.42 to −0.37	0.26	0.785
H‐H' distance	−0.06 ± 4.68	−1.87 to 1.75	−0.0 ± 2.59	−1.07 to 1.06	−0.06	0.978
H‐C3ai distance	7.82 ± 33.79	−8.99 to 24.62	−3.39 ± 11.31	−9.02 to 2.24	11.21	0.196

*Note:* Bold values indicate statistical significance (*p* < 0.05).

A statistically significant increase was observed in the AA–PNS distance (Δ = +2.59 mm, *p* = 0.0442). Similarly, the PNS–p distance increased significantly (Δ = +3.78 mm, *p* = 0.0229).

No significant differences between groups were detected for the velopharyngeal variable ve–pve (Δ = +1.2 mm, *p* = 0.115), nor for the oropharyngeal variables pas–ppas (Δ = +7.76 mm, *p* = 0.27) and ph–pph (Δ = +1.91 mm, *p* = 0.112).

Tongue dimensions (tt–eb length: Δ = +4.7 mm, *p* = 0.127; Th height: Δ = +0.26 mm, *p* = 0.785) and hyoid bone position (H–H′: Δ = −0.06 mm, *p* = 0.978; H–C3ai: Δ = +11.21 mm, *p* = 0.196) did not show statistically significant differences between groups.

## 4 Discussion

This retrospective controlled study evaluated the dento‐skeletal and upper airway effects of a fully intraoral fixed appliance activated with Class III intermaxillary elastics in growing patients with Class III malocclusion. As no statistically significant baseline differences were found between groups for any cephalometric variable, the treated and control groups can be considered comparable at the start of the observation period, so that the between‐group differences observed at T1 are more likely attributable to the treatment than to pre‐existing disparities.

Three main findings emerged. First, the treated group showed a statistically significant improvement of the sagittal intermaxillary relationship as measured by the Wits appraisal, whereas the between‐group difference in the ANB angle did not reach statistical significance. Second, all vertical skeletal parameters remained stable, with no significant between‐group differences for SN^GoGn, FMA or MM angles. Third, airway changes were small and confined to the nasopharyngeal level (AA–PNS) and to the length of the soft palate (PNS–p), with no significant changes at the velopharyngeal or oropharyngeal levels.

The dissociation between the Wits appraisal and the ANB angle deserves careful interpretation, since the two measures, although both designed to describe the sagittal intermaxillary relationship, rely on different anatomical references and are differently affected by craniofacial growth. The ANB angle is constructed from the nasion (N), and any sagittal or vertical displacement of N due to growth of the anterior cranial base, as well as rotational changes of the maxilla and mandible, may artificially modify ANB without reflecting a true change in the intermaxillary relationship. The Wits appraisal, conversely, is measured directly on the occlusal plane and bypasses the cranial base, making it less sensitive to rotational artifacts and generally considered a more reliable indicator of sagittal change in growing patients (Jacobson 1975).

The pattern of changes observed in our sample is consistent with this mechanism: the improvement of the sagittal intermaxillary relationship was accompanied by a stable mandibular position in the treated group (SNB Δ = +0.01°), while a small forward repositioning of the mandible was observed in the untreated controls (SNB Δ = +0.78°), supporting a restraining effect of the Class III elastics on the sagittal expression of mandibular growth. The vertical parameters (SN^GoGn, FMA, and MM) remained stable, indicating that the appliance does not produce the clockwise mandibular rotation classically associated with facemask therapy (Rongo et al. 2017; Cordasco et al. 2014). This is clinically relevant because vertical control is one of the main concerns in the orthopedic management of Class III malocclusion, particularly in patients with a normo or hyperdivergent facial pattern.

When the results of the present study are compared with those reported for other orthopedic protocols, some considerations can be drawn.

Facemask therapy, with or without RPE, has been extensively documented to produce a more pronounced anterior displacement of the maxilla, with a consequent increase in SNA, but this effect is consistently accompanied by clockwise rotation of the mandibular plane and an increase in vertical facial dimensions (Cordasco et al. 2014). The introduction of skeletal anchorage with miniplates or miniscrews further reduces dento‐alveolar side effects, in particular the proclination of the upper incisors, but introduces a surgical step in the treatment protocol (Rutili et al. 2023; De Clerck et al. 2010). The SEC III protocol shares with the present appliance the philosophy of combining intraoral mechanics with Class III elastics, but still requires an extraoral chin‐cup component to control vertical growth (Ferro et al. 2003). The protocol described in the present study, conversely, achieves a sagittal correction and good vertical control through a fully intraoral appliance, without extraoral components and without skeletal anchorage. This may translate into a more favorable compliance, which is historically the main limitation of Class III orthopedic treatment in Inchingolo et al. (2024).

Concerning the dento‐alveolar response, the treated group showed a trend toward labial inclination of the upper incisors (I+/ANS‐PNS, Δ = +2.86°) that did not reach statistical significance, while the lower incisor inclination (IMPA) remained essentially stable (Δ = +1.58°, non‐significant). The direction of the upper incisor change is biomechanically expected with Class III intermaxillary elastics applied to a tooth‐borne appliance, and a comparable pattern has been reported for several intraoral Class III protraction systems (Martina et al. 2020). From a clinical standpoint, the stability of IMPA is a relevant finding, since Class III elastics typically tend to lingualize the lower incisors and to determine an unfavorable dento‐alveolar compensation in the lower arch. These findings suggest that the present appliance is particularly suitable for patients with normally inclined or retroinclined upper incisors, whereas in patients already showing maxillary incisor proclination or bialveolar protrusion, alternative strategies involving skeletal anchorage may be preferable in order to limit further dento‐alveolar compensation at the upper arch.

The airway changes observed in the present study were modest and topographically selective. A statistically significant increase was found at the nasopharyngeal level (AA–PNS, Δ = +2.59 mm) and in the length of the soft palate (PNS–p, Δ = +3.78 mm), while no significant changes were detected at the velopharyngeal (ve–pve) or oropharyngeal levels (pas–ppas, ph–pph). These findings are coherent with the available evidence on the airway effects of Class III orthopedic treatment, which describes small but positive changes concentrated in the naso and velopharyngeal districts after maxillary protraction, and limited or inconsistent effects in the oropharynx (Ming et al. 2018; Baratieri et al. 2011). A plausible mechanism is that the rotational repositioning of the premaxilla and the associated displacement of the posterior nasal spine increase the sagittal dimension of the nasopharyngeal space, while the change in soft palate length may reflect an adaptive response of the soft tissues to the new skeletal configuration. Tongue dimensions and hyoid bone position did not differ significantly between groups, suggesting that the spatial relationships between the tongue, the hyoid bone, and the surrounding structures were not altered by the treatment, in agreement with the absence of significant changes at the oropharyngeal level.

The topographic distribution of the airway changes observed in the present study differs from that reported for other intermaxillary Class III appliances. Kaygisiz et al. evaluated the MTTBA, a removable appliance consisting of maxillary and mandibular splints connected by Class III elastics through a traction bow, and reported an enlargement of the retroglossal portion of the oropharynx after treatment, with no differences in post‐treatment airway shape compared with facemask therapy (Kaygisiz et al. 2022). Conversely, in the present sample, the significant changes were confined to the nasopharyngeal level, with no changes in the oropharynx. Several factors may account for this discrepancy. In the MTTBA, the splints introduce a vertical opening of 5–6 mm in the molar region, and the enlargement of the oropharyngeal airway observed with this appliance has been attributed to the forward movement of the maxilla combined with a posterior (clockwise) rotation of the mandible. The appliance used in the present study, being fixed and band‐anchored, does not modify the vertical dimension of occlusion. Moreover, the study by Kaygisiz et al. did not include an untreated control group, so that the contribution of normal growth to the observed airway changes could not be separated from the treatment effect. Direct comparisons between different intermaxillary approaches, ideally based on three‐dimensional imaging, are needed to clarify whether their different force systems translate into distinct airway responses.

The assessment is based on two‐dimensional cephalometry, which is known to be of limited accuracy for the description of a three‐dimensional airway volume and no functional respiratory data were collected. The present data therefore support the absence of adverse airway effects of the treatment, but they do not allow any conclusion regarding a true functional benefit on breathing or any preventive role on sleep‐disordered breathing.

Several limitations of the present study must be acknowledged. First, the retrospective design and the absence of randomization represent intrinsic methodological limitations that should be considered when interpreting the present results. Second, the present study describes the immediate effects observed at the end of the active treatment phase and does not include a long‐term follow‐up; the stability of the dento‐skeletal changes documented here therefore remains to be confirmed by future investigations. Third, the airway assessment was performed on two‐dimensional lateral cephalograms, which represented the standard imaging modality available in the retrospective setting of this study but constitute an intrinsic limit when describing a three‐dimensional anatomical district. Fourth, the control group was drawn from a historical longitudinal growth sample. Although the controls were matched for skeletal disharmony, sex, chronological age and observation interval and no baseline differences were found, the comparison with a historical sample may be affected by secular trends in craniofacial growth.

Within the limits described above, the present study suggests that a fully intraoral fixed appliance activated with Class III intermaxillary elastics can represent a valid early treatment option in growing patients with Class III malocclusion, particularly in those with a normo‐ or hypodivergent facial pattern and with normally inclined or retroinclined upper incisors. The protocol achieves a clinically relevant improvement of the sagittal intermaxillary relationship together with a good vertical control. Patients with marked maxillary incisor proclination, bialveolar protrusion or severely hyperdivergent facial patterns may be less suitable candidates. Future research should ideally include prospective randomized controlled trials with concurrent and ethnically homogeneous control groups, long‐term follow‐up extending beyond the pubertal growth peak to characterize the stability of the correction and direct comparisons with other treatment options in order to better define the indications, the limits and the relative effectiveness of this fully intraoral approach.

## 5 Conclusion

Within the limits of its retrospective design, the present controlled study suggests that a fully intraoral fixed appliance activated with Class III elastics may be associated with an improvement of the sagittal maxillo‐mandibular relationship in growing Class III patients, as documented by a statistically significant between‐group difference for the Wits appraisal. The between‐group difference in the ANB angle did not reach statistical significance. Vertical facial parameters did not show significant changes between groups, suggesting a favorable vertical behavior during treatment. A non‐significant trend toward upper incisor proclination was observed in the study group, consistent with the expected biomechanics of Class III elastics.

Airway modifications, although limited and topographically selective, consisted of small increases at the nasopharyngeal level and in the length of the soft palate, with no significant changes at the velopharyngeal or oropharyngeal levels.

Overall, the present findings provide preliminary evidence that the intraoral Class III elastic protocol evaluated in this study could represent a possible early treatment option, with a favorable sagittal effect and without significant adverse changes in vertical facial dimensions. Given the retrospective design of the study and the absence of long‐term follow‐up, these results should be interpreted with caution and considered as hypothesis‐generating. Prospective controlled studies with larger samples, three‐dimensional airway assessment and long‐term follow‐up are needed to confirm and extend the present findings.

## Author Contributions

**Michele d'Attilio:** conceptualization, review. **Gianluca Rosa:** writing. **Salvo Luca Gallo:** data curation, statistics. **Giuliano Ascani:** data curation. **Antonino Peluso:** conceptualization, data curation, statistics, writing, review.

## Funding

The authors have nothing to report.

## Conflicts of Interest

The authors declare no conflicts of interest.

## Data Availability

The data that support the findings of this study are available from the corresponding author upon reasonable request.
